# Key outcomes for reporting in studies of pregnant women with multiple long-term conditions: a qualitative study

**DOI:** 10.1186/s12884-023-05773-5

**Published:** 2023-08-01

**Authors:** Siang Ing Lee, Stephanie Hanley, Zoe Vowles, Rachel Plachcinski, Amaya Azcoaga-Lorenzo, Beck Taylor, Catherine Nelson-Piercy, Colin McCowan, Dermot O’Reilly, Holly Hope, Kathryn M. Abel, Kelly-Ann Eastwood, Louise Locock, Megha Singh, Ngawai Moss, Sinead Brophy, Krishnarajah Nirantharakumar, Shakila Thangaratinam, Mairead Black

**Affiliations:** 1 Institute of Applied Health Research, University of Birmingham, Birmingham, UK; 2 Guy’s and St. Thomas’ NHS Foundation Trust, London, UK; 3 Patient and public representative, London, UK; 4 Division of Population and Behavioural Sciences, School of Medicine, University of St Andrews, St Andrews, UK; 5 Centre for Public Health, Queen’s University of Belfast, Belfast, UK; 6 Centre for Women’s Mental Health, Faculty of Biology Medicine & Health, The University of Manchester, Manchester, UK; 7 Greater Manchester Mental Health NHS Foundation Trust, Manchester, UK; 8 St Michael’s Hospital, University Hospitals Bristol NHS Foundation Trust, Bristol, UK; 9 Health Services Research Unit, School of Medicine, Medical Science and Nutrition, University of Aberdeen, Aberdeen, UK; 10 Data Science, Medical School, Swansea University, Swansea, UK; 11 WHO Collaborating Centre for Global Women’s Health, Institute of Metabolism and Systems Research, University of Birmingham, Birmingham, UK; 12 Department of Obstetrics and Gynaecology, Birmingham Women’s and Children’s NHS Foundation Trust, Birmingham, UK; 13 Aberdeen Centre for Women’s Health Research, School of Medicine, Medical Science and Nutrition, University of Aberdeen, Aberdeen, UK

**Keywords:** Multimorbidity, Multiple chronic conditions, Multiple long-term conditions, Pregnancy, Maternity, Outcome, Core outcome set

## Abstract

**Background:**

Maternal multiple long-term conditions are associated with adverse outcomes for mother and child. We conducted a qualitative study to inform a core outcome set for studies of pregnant women with multiple long-term conditions.

**Methods:**

Women with two or more pre-existing long-term physical or mental health conditions, who had been pregnant in the last five years or planning a pregnancy, their partners and health care professionals were eligible. Recruitment was through social media, patients and health care professionals’ organisations and personal contacts. Participants who contacted the study team were purposively sampled for maximum variation. Three virtual focus groups were conducted from December 2021 to March 2022 in the United Kingdom: (i) health care professionals (n = 8), (ii) women with multiple long-term conditions (n = 6), and (iii) women with multiple long-term conditions (n = 6) and partners (n = 2). There was representation from women with 20 different physical health conditions and four mental health conditions; health care professionals from obstetrics, obstetric/maternal medicine, midwifery, neonatology, perinatal psychiatry, and general practice. Participants were asked what outcomes should be reported in all studies of pregnant women with multiple long-term conditions. Inductive thematic analysis was conducted. Outcomes identified in the focus groups were mapped to those identified in a systematic literature search in the core outcome set development.

**Results:**

The focus groups identified 63 outcomes, including maternal (n = 43), children’s (n = 16) and health care utilisation (n = 4) outcomes. Twenty-eight outcomes were new when mapped to the systematic literature search. Outcomes considered important were generally similar across stakeholder groups. Women emphasised outcomes related to care processes, such as information sharing when transitioning between health care teams and stages of pregnancy (continuity of care). Both women and partners wanted to be involved in care decisions and to feel informed of the risks to the pregnancy and baby. Health care professionals additionally prioritised non-clinical outcomes, including quality of life and financial implications for the women; and longer-term outcomes, such as children’s developmental outcomes.

**Conclusions:**

The findings will inform the design of a core outcome set. Participants’ experiences provided useful insights of how maternity care for pregnant women with multiple long-term conditions can be improved.

**Supplementary Information:**

The online version contains supplementary material available at 10.1186/s12884-023-05773-5.

## Background

Women with long-term conditions are at higher risk of adverse pregnancy outcomes [1, 2]. Pregnancy can also impact on women’s underlying long-term conditions [3]. These challenges are likely to be multiplied in women who have two or more long-term conditions, also known as multimorbidity or multiple long-term conditions [4]. They may have to take multiple medications [5] or attend appointments with different health care teams to manage their multiple long-term conditions [6]. Recent studies suggest that maternal multiple long-term conditions are associated with higher risk of adverse outcomes such as preterm birth [7, 8]. This is significant as one in five pregnant women has multiple long-term conditions in the United Kingdom (UK) [9]. Current health care systems and guidelines are configured for single health conditions [10]. Therefore maternal multiple long-term conditions present a unique challenge to pregnancy and is a priority for maternity research [11].

An outcome is a measurement or observation used to assess the effect of an intervention or an exposure (in this case maternal multiple long-term conditions) to the health and well-being of the population of interest [12, 13]. The MuM-PreDiCT consortium is developing a core outcome set for studies of pregnant women with multiple long-term conditions [14]. This minimum set of outcomes is recommended to be reported in all studies in this field to enable comparison between studies and combining of information in systematic reviews and meta-analyses [15]. To ensure its relevance, the core outcome set is being developed with multiple stakeholders, including pregnant women with multiple long-term conditions and health care professionals.

The study protocol for the core outcome set has been reported elsewhere, [14] but briefly it involves a four stage process: systematic literature search and focus groups to generate an initial list of outcomes, followed by prioritisation through Delphi surveys and a consensus setting meeting [14].

Systematic reviews of outcomes reported in previous literature may not represent the views of key stakeholders, especially service users [13]. Our systematic literature search did not identify qualitative studies of pregnant women with multiple long-term conditions. The Core Outcome Measures for Effectiveness Trials (COMET) Handbook recommends supplementing the initial list of outcomes with qualitative studies involving key stakeholders [13]. The words participants used to describe their views and experiences can subsequently be used to label and explain outcome items in the Delphi surveys [13, 16].

This focus group study aims to explore outcomes that women, partners and health care professionals feel should be reported in all studies of pregnant women with multiple long-term conditions. The findings will inform the design of a Delphi survey for a core outcome set for studies or pregnant women with multiple long-term conditions.

## Methods

### Study design

Interviews and focus groups have been used as qualitative methods to inform core outcome sets [16]. As the experience and outcomes of pregnancy may vary depending on the women’s unique combination of health conditions, we chose to conduct focus groups for synergistic discussions [16].

### Inclusivity statement

Where the words ‘women’, ‘maternal’, or ‘mother’ are used, these also include people who do not identify as women but have been pregnant, planning to be pregnant or have given birth.

### Inclusion criteria

Participants were eligible for the focus groups if they were women with two or more pre-existing long-term physical or mental health conditions, who have been pregnant in the last 5 years or are planning a pregnancy; their partners; and health care professionals who look after pregnant women with multiple long-term conditions and their children. Participants had to be able to converse in English and based in the UK.

### Recruitment

We planned to conduct three focus groups, one for health care professionals, one for women, and one for women with or without their partners. After discussion with our patient and public involvement (PPI) advisory group, we aimed to recruit eight participants per focus group to facilitate optimal discussion and to account for dropouts. The PPI advisory group also recommended inviting partners to attend alongside their pregnant partner, instead of a focus group for partners only. This would help focus the discussion on outcomes relevant to studies of pregnant women with multiple long-term conditions.

Recruitment and sampling was guided by a sampling matrix prespecified in the core outcome set protocol, based on physical or mental health conditions, ethnicity, under-served populations and specialties of health care professionals [14]. We additionally aimed for representation from all four devolved nations in the UK and partners.

Study adverts were shared through social media platforms (Facebook, Twitter, Instagram and websites) of charities and organisations for patients, pregnancy, mothers, and health care professionals, and with personal contacts of the multidisciplinary research team. The recruitment campaign took place in October 2021 for health care professionals and January 2022 for women and partners, and lasted for two to three months. Potential participants contacted the research team directly and were provided with the participant information sheets. They completed a sampling questionnaire which iteratively informed the recruitment strategy [14]. We then undertook maximum variation purposive sampling from the pool of eligible potential participants [16].

### Data collection

The initial topic guide was developed based on the study aim and previous qualitative studies for core outcome set development in obstetrics [16–18]. This was then reviewed by and pilot tested (test run of a group discussion) with our patient and public involvement (PPI) advisory group. The discussion in the pilot test focused on maternity care experiences and proposed solution. In order to efficiently draw out discussions on outcomes, the PPI advisory group advised that the topic guide was simplified to an open question of what outcomes are important to stakeholders, and included a case vignette to illustrate what is an outcome. The topic guide was then revised based on their feedback (Supplementary Material 1).

Three focus groups were conducted from December 2021 to March 2022, one for each of the following groups:

1. health care professionals,
2. women with multiple long-term conditions, and
3. women with multiple long-term conditions with or without their partners.

Due to difficulties with face-to-face meetings during the COVID-19 pandemic, the focus groups were conducted virtually using Zoom and audio recorded.

The lead facilitator (SIL) is a female doctoral student, public health specialist trainee and qualified as a general practitioner. She has previously undertaken qualitative data analysis and training in qualitative research. The supporting team included researchers with expertise in qualitative research in health service, public health and maternity.

The health care professionals’ focus group was planned for one hour to increase participation rate, based on feedback from potential participants. There were two facilitators: one led the discussion whilst one monitored the chat function. To build rapport with the participants, the lead facilitator shared her clinical background.

The two women and partners’ focus groups each lasted two hours. There were three facilitators, the additional clinical facilitator was designated to provide support should participants become distressed. Women and partners were emailed a £25 e-voucher each for reimbursement. The lead facilitator shared her medical history (long-term condition) and characteristics of under-served populations (physical disability and ethnic minority) with the participants. To encourage participants to speak freely, the facilitators did not share their clinical background. After each focus group, the facilitators debriefed and reflected on their initial impressions.

### Analysis

Thematic analysis was conducted with an inductive approach [19]. The analysis focused on research outcomes discussed or inferred by participants. The stages of pregnancy were used as a prompt to facilitate the focus group discussions. Therefore, we pre-specified that themes (outcomes) will be provisionally categorised by the different stages of pregnancy (before, during and after pregnancy) and by maternal and children’s outcomes.

The audio recordings of the focus groups were transcribed verbatim by SIL. This allowed for familiarisation with the data. Two researchers coded the transcripts independently, the initial codes were collated into potential themes using NVivo 12 and Microsoft Word. The codes and themes / outcomes were then compared and discussed. Themes identified from the focus groups of health care professionals and women/partners were compared and contrasted in tabular form.

Further checking was conducted by a multidisciplinary team, including MB (obstetrician), ZV (midwife) and RP (woman’s representative) who read the transcripts and reviewed the developed themes. The key themes with extracted quotes were presented to our PPI advisory group, with their opinions sought on key queries, especially on the labelling of themes. This approach helped maintain researcher reflexivity [20] and enriched the analysis. The outcomes identified in the focus groups were then compared with the list of outcomes identified in a systematic literature search.

### Patient and public involvement

The PPI advisory group advised on the study design and recruitment strategy, design of the recruitment materials (e.g. participant information sheets and study poster) and shared the study advert through their networks. They pilot tested the topic guide and was involved in interpreting the data analysis. A PPI co-investigator (RP) reviewed the anonymised transcripts against the key themes identified. The final manuscript was reviewed by PPI co-investigators (RP and NM).

## Results

### Characteristics of study participants

Supplementary Material 2 presents the participant flow chart. Nineteen health care professionals expressed interest and were eligible, 10 were available on the scheduled focus group date and were invited to participate. Three health care professionals who expressed interest thereafter were kept on the waiting list and all were eventually invited to participate as five original participants could not attend. Twenty-five women expressed interest and were available on the focus group dates, 18 were invited to participate based on the sampling frame, subsequently six could not attend. Overall, eight health care professionals, 12 women and two partners participated in one of the three focus groups. Table 1 presents the characteristics of study participants for each focus group. There was representation from health care professionals from obstetrics, obstetric/maternal medicine, midwifery, neonatology, perinatal psychiatry, and general practice; and women with 20 different physical health conditions and four mental health conditions.

**Table 1:** Characteristics of study participants

Characteristics	Focus group 1	Focus group 2	Focus group 3
**Stakeholder groups**	Health care professionals	Women with multiple long-term conditions and experience of pregnancy in the last 5 years / planning a pregnancy	Women with multiple long-term conditions and experience of pregnancy in the last 5 years / planning a pregnancy, and their partner
**Time period**	December 2021	February 2022	March 2022
**Total number of participants, n**	8	6	8 (4 women attended solo, 2 women attended with their partners)
**Specialty / job role, n**			
General practitioner	1	-	-
Maternal medicine subspecialist	1	-	-
Midwife	2	-	-
Neonatologist	1	-	-
Obstetrician	1		
Obstetric medicine specialist (general physician)	1	-	-
Perinatal psychiatrist	1	-	-
**Pregnancy, n**			
Pregnant in the last 5 years	-	4	3
Planning a pregnancy	-	1	2
Pregnant in the last 5 years and planning a pregnancy	-	1	1
**Total number of self-reported long-term conditions**			
Range	-	2 to 6	2 to 8
Median (IQR)	-	3 (2 to 3)	4 (2 to 4)
**Physical health conditions**			
Arrhythmia	-	-	1
Asthma	-	-	1
Cardiomyopathy	-	-	1
Coeliac disease	-	1	-
Degenerative disc disease	-	1	-
Diabetes mellitus	-	2	1
Factor V Leiden	-	1	-
Fibromyalgia	-	-	1
Functional neurological disorder	-	1	-
Gastroesophageal reflux disease, sphincter of Oddi dysfunction, gastritis	-	-	1
Hypermobile Ehlers-Danlos syndrome	-	1	-
Human immunodeficiency virus infection	-	1	1
Hypertension	-	1	1
Inflammatory bowel disease, ulcerative proctitis	-	1	1
Irritable bowel syndrome	-	-	1
Myofascial pain syndrome	-	1	-
Orthostatic hypotension	-	1	-
Polycystic ovarian syndrome	-	1	-
Psoriasis	-	-	2
Psoriatic arthritis	-	-	2
**Mental health conditions**			
Anxiety	-	2	3
Bipolar affective disorder	-	-	1
Complex post-traumatic stress disorder	-	1	1
Depression	-	3	2
**Under-served population**	-	Carer, disabled/deaf/blind, LGBTQ+, migrant, victims of domestic abuse.	LGBTQ+, refugee
**Rural, n**	-	1	5
**Education level, n**	-		
GCSE	-	-	1
A levels	-	1	1
Diploma		-	2
College	-	1	-
University	-	4	4
**Ethnicity, n**			
Asian	3	1	-
Black, Caribbean or African	1	2	2
White	2	3	6
Mixed / multiple ethnic groups	1	-	-
Other	1	-	-
**Age in years**			
Range	32 to 64	28 to 44	22 to 49
Median (IQR)	54 (47 to 55)	33 (31 to 41)	38 (28 to 40)
**Country, n**			
England	7	3	6
Northern Ireland	-	-	2
Scotland	1	3	-

GCSE: general certificate of secondary education, IQR: interquartile range, LGBTQ+: lesbian, gay, bisexual, transgender, queer and others. NB: Maternal medicine specialists are obstetricians who subspecialise in maternal medicine; obstetric medicine specialists are internal medicine physicians who subspecialise in care of the pregnant women

### Thematic categories

Table 2 presents the coding tree consisting of thematic categories, themes / outcomes and subthemes. Five thematic categories were identified: (i) Care Outcomes, (ii) Clinical Outcomes, (iii) Role as mothers or parents, (iv) Other outcomes, and (v) Consideration for future studies. An overview of the thematic categories is presented here with selected quotes, with supplementary quotes in Supplementary Material 3.

**Table 2:** Coding tree: Thematic categories, themes /outcomes and subthemes

Thematic categories	Themes /outcomes	Subthemes
Care outcomes	Preconception support	Uptake of preconception support Quality of preconception counselling Preconception counselling on medications
	Interventions in pregnancy	Limited options Analgesia
	Postnatal and long-term care	Quality of postnatal support Length of postnatal support Postnatal support for raising a child Emotional support Support for family
	Breastfeeding support	
	Skin-to-skin	
	Quality and experience of care	
	Holistic care / multidisciplinary coordination of care	
	Shared care decision	
	Continuity of care	Information being passed on
		Seeing the same health care professionals
	Social and peer support	
	Information provision for preparedness	Informed of care
		Informed of potential risks
		Informed of support / services available
		Informed for self care
	Birth experience	
	Accessibility of services	Physical barriers
		Social barriers
		Communication barriers
		Travel distances
	Health care professional (HCP)s’ skills and knowledge	
	HCPs’ knowledge of the woman	
	HCPs’ attitude towards the woman	
	Hospitals’ facilities and services	
	Personalised care	
	Consistency of care	
	Expectation of care and outcomes	
	Separation of mother from newborn baby	
	Admission to neonatal unit	
	Number of appointments	
	Length of hospitalisation	
	Number of admissions during pregnancy	
Clinical outcomes		
	Fertility	
	Maternal death	
	Impact on long-term conditions	
	Types of birth	
	Miscarriage	
	Birth injuries	
	Haemorrhage	
	Blood pressure	
	Perinatal mental health	Postnatal depression
		Impact on pre-existing mental illness
		Impact of mental health on physical health
		Impact of physical health on mental health
		Emotional and mental well-being
		Experience of perinatal mental health support
	Recovery time	
	Development of new health conditions	
	Long-term cardiovascular outcomes	
	Engagement with health behaviours	
	Change in medications	
	Compliance with medications	
	Quality of life	
	Timing of birth (preterm)	
	Baby’s lung development (respiratory distress syndrome)	
	Infant mental health	
	Child’s death	Neonatal mortality
		Infant mortality
	Baby’s condition at birth	
	Birth defect	
	Birth weight	
	Impact of medication in pregnancy on child	
	Neonatal intervention	
	Inheritance of mother’s conditions	
	Baby’s growth	
	Developmental outcomes (child)	
	Metabolic syndrome (child)	
	Neonatal morbidity	Neonatal cardiovascular function
		Neonatal metabolic control
		Neonatal jaundice
		Baby’s physiology
Role as mothers / parents	Ability to breastfeed	
	Establishing feeding	
	Maternal guilt	
	Pressure in maternal role	
	Parent and infant bonding	
Other outcomes	Impact on partner	Partner’s caring role
		Support for partner
		Involvement of partner
		Partner’s mental well being
	Financial implications	
	Participation in society (child)	
Considerations for future studies	Impact of ethnicity	
	Framing outcomes positively	
	Focus on experiential outcomes	

### Care outcomes

Participants highlighted stages of pregnancy where input from health and social care professionals were important. Preconception counselling was important to plan whether women have to change or stop medications they take for their long-term conditions. Women and health care professionals felt that postnatal support should be longer than the conventional six weeks given the complexity of women’s multiple long-term conditions. Women may need support looking after their newborn baby, whilst managing their multiple long-term conditions that may have been adversely impacted by the pregnancy. Key care outcomes were whether the relevant components of care were provided and of good quality.

Specific components of care were discussed at length. Examples relevant to women with multiple long-term conditions included: multidisciplinary coordination of care, holistic care and continuity of care. Health care professionals said that women want to know whether they or their baby will need to have more appointments or tests than routine care. Women described the burden of having to attend multiple appointments with different specialties and want more coordinated care. Continuity of care included transfer of information as women and their baby transitioned through different teams (e.g., specialist team to general team) and different stages of pregnancy. They valued seeing the same health care professionals throughout pregnancy:

*“I had a fantastic consultant…he was there throughout ...an advocate who knew all my health conditions, he led on one of them, but he knew the others and linked up with my other doctors.” (FG2, women [W]4)*

Feeling informed of their care and conditions was an outcome that was frequently discussed. Women and partners valued honest communication of potential risk to their pregnancy and baby. They want to be informed of: what is happening during birth, the side effects of medication in pregnancy, actions they can take for self-care, and support or services available for their specific needs. Being sufficiently informed was crucial in helping them mentally prepare to face adverse outcomes.

*“…I needed to prepare myself, psychologically for the possibility that the baby might not be able to stay with me [after birth]…having that information before…made it easier for me to manage ...” (FG3, W4)*

Women shared accounts of when they experienced discrimination due to health care professional’s attitudes, to illustrate respectful care as an important outcome.

*“…they said…how are you going to manage to look after your child…because disabled women are seen as…not basically being suitable for having children that we just get completely bypassed.” (FG2, W2)*

Having maternity services that were accessible was an important structural measure of care. One participant shared experiences of encountering physical (e.g., lack of wheelchair access), social (e.g., domestic violence) and communication barriers (e.g., lack of sign language interpreters).

Health care professionals and women spoke about women’s desire to have minimal intervention during pregnancy and to their baby. However, the care needs arising from pregnant women’s multiple long-term conditions may limit their birthing and care options. Women described the devastation of being separated from their newborn who required additional support after birth. These are outcomes that health care professionals would like to see being studied so they can counsel women. Despite the limitations of options, feeling involved in their care decision is an important outcome, as one participant shared her experience when this did not happen:

*“…the consultant…goes, we just had a meeting and we decided that you cannot have a [type of birth]…I go…all…of you should be in here now, because you made decisions without me.” (FG2, W1)*

Women spoke about the importance of measuring their experience of care, throughout the pregnancy and specifically during birth, and whether there was any birth injuries. Despite being involved in their birth plans, non-adherence by the care team can lead to women having negative birth experience. One disabled participant spoke about how difficult births may not relate to the obstetric factors, but stemmed from the women not being listened to.

*“…the main outcomes that need to be looked at are satisfaction with experience…birth satisfaction…I know a lot of people who’ve been through some very difficult births and it’s not related to the difficulties. It’s related to…not being listened to, not being allowed adequate pain relief…not having their physical or mental health concerns taken into account.” (FG2, W2)*

### Clinical outcomes

Participants spoke about clinical outcomes such as maternal death, stillbirth, infant mortality and preterm birth. Clinical outcomes that were specific to pregnant women with multiple long-term conditions included the impact of pregnancy on the women’s long-term conditions (e.g., improvement in or worsening pain in inflammatory arthritis), the development of new health conditions (e.g., type 2 diabetes), and whether children inherited their mothers’ long-term conditions.

One area of particular interest was the impact of medication in pregnancy. Availability of information on medication safety in pregnancy was important to women. Health care professionals felt it was important to measure the extent of non-adherence to medications as some women may stop their regular medications when pregnant or breastfeeding because of concerns about how the medications may affect their baby. Women struggled to articulate specific outcomes they were concerned about, but mentioned miscarriage, birth defects and baby’s condition at birth. Health care professionals emphasised the importance of balanced discussion with women:

*“…it’s helpful to know the effects of…untreated…disease on the outcomes of the children so that you can weigh up the benefits and the risks of taking medication...” (FG1, health care professionals [HCP]1)*

Perinatal mental illness, emotional and mental wellbeing, and satisfaction with perinatal mental health support were identified as important outcomes. Health care professionals and women discussed how mental and physical health conditions are interlinked and can influence each other. They spoke about the emotional stress that women experienced long after the birthing event and hence the importance of receiving good perinatal mental health support. One participant highlighted that mental illness is a taboo in some ethnic minority groups, which may impede access to diagnoses and support. Women shared their experience of not having satisfactory perinatal mental health support:

*“I’ve had serious and complicated mental health issues, since our…[child] was born and I still have never been referred to community psychiatry. I got a very reluctant acceptance through [the] perinatal mental health [team]…” (FG3, W2)*

### Role as mothers or parents

Both health care professionals and women participants spoke about the pressure women felt to be the perfect mother and to breastfeed. Where there were adverse child outcomes, health care professionals and women participants spoke of the guilt some pregnant women experienced, as they attributed the adverse child outcomes to their own long-term conditions or the medications they have to take. Women spoke about how circumstances around birth may disrupt parent (including the father) and infant bonding. Health care professionals discussed that mother and infant bonding and the ability to engage with parental roles could be outcomes to measure when evaluating an intervention. For example, in perinatal psychiatry:

“*destabilization of [the women’s] mental health [can lead to] potential disruption to mum, to baby, to the bond…[disruption to] establishing feeding…” (FG1, HCP8)*.

### Other outcomes

One of the key themes in this category was how the pregnancies of women with multiple long-term conditions impacted on their partner. Women described the carer role that their partner take on, to help them with their activities of daily living or managing their long-term conditions. Partners want to be informed of actions they can take to look after mother and baby. Women spoke of the emotional stress their partner experienced during the childbirth process, as they were fearful of what might happen to the women. Partners shared contrasting experience of their involvement in the care of the pregnant women.

*“…We’re just the people who drive them there… we’re constantly left where we may have to pick up the pieces of whatever has happened…I’ve just been ignored by doctors.” (FG3, Partner [P]1)*

*“…the nurses were keeping me well assured about what was going on…Even though you were freaking out… you’re always well informed…” (FG3, P2)*

Consequently, whether partners felt they were involved and supported, in addition to their emotional and mental wellbeing were identified as important outcomes.

### Considerations for future studies

Health care professionals raised some considerations for future studies of pregnant women with multiple long-term conditions. This included assessing how outcomes may differ by ethnicity.

One health care professional spoke about framing outcomes in a positive way:

*“…the impacts that we’re considering for multiple morbidities are always negative…I wonder if we could have positive impacts. Having had a baby, people feel so much happier and better. They didn’t think it was going to go well, but actually it did…” (FG1, HCP3)*

Health care professionals discussed that maternal and children’s outcomes will vary greatly, depending on the women’s long-term conditions. Sometimes women’s outcome expectations may not be achievable. Therefore, core outcomes for studies of pregnant women with multiple long-term conditions should reflect experiences and not just binary outcomes:

*“…I may recommend you not to get pregnant, because your risk…is so high, you may choose to get pregnant, but the satisfaction should be… you felt supported… whether your expectation has been met or not.” (FG1, HCP4)*

### Outcomes by stakeholder groups

Table 3 tabulates the 63 outcomes by stakeholder groups. These were presented by maternal (43 outcomes) and children’s outcomes (16 outcomes), health care utilisation (4 outcomes) and by the stages of pregnancy.

**Table 3:** Identified outcomes presented by stakeholder groups

Stages of pregnancy		Women / partner and health care professionals	Women / partners only	Health care professionals only
**Maternal outcomes, n = 43**	**Before pregnancy** **n = 2**	Fertility Preconception care	-	-
	**During pregnancy** **n = 8**	Maternal death Impact on long-term conditions Types of birth	Miscarriage Birth injuries	Interventions in pregnancy Haemorrhage Blood pressure
	**After pregnancy** **n = 13**	Postnatal and long-term care Perinatal mental health Ability to breastfeed Pressure in maternal role Maternal guilt Parent and infant bonding	Breastfeeding support Skin-to-skin Recovery time Development of new health conditions	Long-term cardiovascular outcomes Engaging with healthy behaviour Establishing feeding
	**All stages of pregnancy** **n = 20**	Quality and experience of care Change in medication Holistic care / multidisciplinary coordination of care Shared care decision Continuity of care Social and peer support Information provision for preparedness	Birth experience Accessibility of services Health care professionals’: - Knowledge and skills - Knowledge of the woman - Attitude towards the woman Hospitals’ facilities / services Personalised care Consistency of care Impact on partner	Financial implications Expectation of care and outcomes Compliance with medication Quality of life
**Children’s outcome** **n = 16**		Timing of birth (preterm birth) Separation of mother from newborn baby Baby’s lung development (respiratory distress syndrome) Infant mental health Child’s death Baby’s condition at birth Birth defect Birth weight / macrosomia Impact of medication in pregnancy	Neonatal intervention Inheritance of mother’s condition	Baby’s growth Developmental outcomes Metabolic syndrome Neonatal morbidity Participation in society
**Health care utilisation** **n = 4**		Admission to neonatal unit Number of appointments	-	Length of hospitalisation Number of hospital admission during pregnancy

### Comparison with outcomes reported in the literature

For the core outcome set development, our systematic literature search [14] included two core outcome sets for maternity care, pregnancy and childbirth, [21, 22] one core outcome set for multiple long-term conditions [23] and 26 studies of pregnant women with multiple long-term conditions, [7, 8, 24–47] which reported 185 outcomes. When mapped to these outcomes reported in the literature, this focus group study identified an additional 28 outcomes (Table 4).

**Table 4:** Additional outcomes identified in the focus groups

**Maternal outcomes** ***Before pregnancy*** 1. Fertility 2. Preconception care ***During pregnancy*** 3. Impact on long-term conditions 4. Miscarriage ***After pregnancy*** 5. Development of new health conditions 6. Long-term cardiovascular outcomes 7. Maternal guilt 8. Parent and infant bonding 9. Postnatal and long-term care 10. Pressure in maternal role 11. Recovery time ***All stages of pregnancy*** 12. Accessibility of services 13. Change in medication 14. Consistency of care 15. Continuity of care 16. Expectation of care and outcomes 17. Holistic care / multidisciplinary coordination of care 18. Hospital’s facilities / services 19. Impact on partner 20. Personalised care 21. Social and peer support **Children’s outcome** 22. Baby’s growth 23. Impact of medication in pregnancy on child 24. Infant mental health 25. Inheritance of mother’s condition 26. Metabolic syndrome 27. Participation in society 28. Separation from newborn baby	

## Discussion

### Main findings

We explored research outcomes that are important to women with multiple long-term conditions who have been pregnant or who are planning a pregnancy, partners and health care professionals. In comparison to outcomes identified from a systematic literature search, our focus groups identified an additional 28 outcomes. Outcomes considered important were generally similar across stakeholder groups. Women emphasised on outcomes related to care processes and wanted to feel informed of the risks to their pregnancy, their health conditions and their baby. Partners said it was important to be informed of risks to the pregnant women and what they can do to look after mother and baby, and to feel involved in their care. Health care professionals additionally prioritised non-clinical outcomes, such as quality of life and financial implications for the women; and longer-term outcomes, especially for children, such as developmental outcomes.

### Comparison with the literature

#### Medication in pregnancy

Medication in pregnancy may be a particular challenge for pregnant women with multiple long-term conditions as they may have to take many different medications [48]. In the focus groups, women specifically wanted information on medication safety in pregnancy and were concerned about the general risks on their babies. This is consistent with findings from studies of pregnant women with single long-term conditions [49–54]. However, within the focus groups, it was difficult to elicit specific outcomes that women were worried about for their children in relation to medication use. Other qualitative studies about medication in pregnancy have reported that women are concerned specifically about the effect of medication on fetal development, congenital anomalies and developmental disability [51, 53–55]. Our health care professional participants expressed concerns about attributing observed adverse outcomes to fetal exposures in utero; or to suboptimal management of maternal health conditions.

#### Aspects of care

Previous literature on single long-term condition in pregnant women reveal several aspects of care that were also important to our participants. Women with inflammatory arthritis spoke about needing to time their conception and to adjust their medications, [51] highlighting the importance of preconception planning.

Earlier work on women with long-term conditions described their feeling of abandonment by health care professionals after giving birth [6]. They felt that their long-term conditions affected their ability to look after their babies [51–53]. The need for longer postnatal support was discussed both by women and health care professionals.

Studies suggest that women with long-term conditions may be more likely to develop perinatal mental illness [28, 56]. Here, health care professionals discussed the importance of providing perinatal mental health support because of their understanding of the link between maternal mental illness and maternal bonding with the baby.

Women with long-term conditions described their experience of fragmented clinical care, meaning they took on the role of relaying information between different health care professionals [6]. Women also shared experiences of feeling discriminated against by health care professionals through the language used and how they were treated. In Rebić et al’s systematic review on key challenges of pregnancy with inflammatory arthritis, women described facing ‘judgement’ from the community and health care professionals for their pregnancy intention and ability to fulfil parental responsibility [51].

#### Single versus multiple long-term conditions

Our focus group did not include pregnant women with no or single long-term conditions for comparison with pregnant women with multiple long-term conditions. However, the findings in this study is likely to be common to all women who have been pregnant or given birth, with or without multiple long-term conditions. This is reflected by the overlap of findings with existing core outcome sets for all pregnancies in general [21, 22] and with studies of pregnant women with single condition [49–54]. A few of our findings are specific to pregnant women with multiple long-term conditions, such as quality of life, number of appointments and hospital admission (treatment burden), and development of new long-term conditions, as observed in the core outcome set for multimorbidity [23].

There is currently no qualitative study specific to pregnant women with multiple long-term conditions. Hansen et al. interviewed pregnant women with one or more long-term conditions in Denmark [6]. Although just under half of the study participants had two or more long-term conditions, it was not possible to disentangle the findings for those with (two or more conditions) and without (single conditions) multiple long-term conditions. Their experience is likely to be similar as even pregnant women with single condition still have to see multiple health care professionals (e.g. obstetrics, anaesthetist, and specialist for their long-term conditions) and balance the risk to their long-term conditions, pregnancy, and unborn child. However, the complexity and treatment burden is likely to be heighten when there are more than one long-term conditions to account for in the pregnancy care plan.

### Strengths and limitations

#### Representation of stakeholders

This focus group study involved a wide range of stakeholders, including health care professionals from different specialties, women with different long-term conditions, both who have had experience of pregnancy, childbirth and those who are planning a pregnancy, and their partners. In line with other qualitative studies in core outcome set development, we have included more service users than health care professionals to identify outcomes [57]. This is to ensure outcomes preferred by service users are included in subsequent prioritisation methods [57].

This study included the perspective of partners, who may be a family member or take on the role of a carer. Previous studies have also included the perspectives of carers as ‘involved witnesses’ [16]. The presence of partners may enhance or inhibit the disclosure by women participants and vice versa. To partially account for this, one focus group was dedicated to women participants only. We did not conduct a focus group for partners only, and therefore may not have fully captured their views. However, in the focus group where two partners participated, there were discussions specifically around the impact of pregnancies of women with multiple long-term conditions, on partners and the family unit.

A key limitation of this study is the small sample size. As this study is part of a mixed-method core outcome set development and wider work of the MuM-PreDiCT consortium, within the available resources and project timeline, we only conducted three focus groups. The number of focus groups was not guided by data saturation. Despite using a maximum variation sampling strategy, it was not possible to have representation from all health conditions. It was also challenging to recruit partners. Therefore, it is possible that we have not captured all outcomes that are important to key stakeholders.

However, the literature search has already identified a long comprehensive list of reported outcomes. It included core outcome sets of pregnancy, childbirth and maternity care in general, [21, 22] which were developed with service users and overlapped with many outcomes identified in this focus group. In addition, there will be opportunities for participants to suggest missing outcomes in the Delphi survey.

Participants were limited to those that could converse in English and in the UK. This limits the transferability of the study findings to non-English speaking pregnant women within the UK and other countries with different health care system, especially low-middle income countries.

#### Data collection

Qualitative methods used to inform core outcome set development included interviews, focus groups and secondary analysis of archived qualitative studies [16, 17]. In this focus group study, we observed that participants were empowered to share their feelings and experiences after listening to other participant’s stories, especially those with under-served characteristics or negative care experiences. However, some participants may find focus groups to be intimidating and inhibitive [16]. To overcome this, we harnessed the advantages of an online platform. Two participants chose to communicate using the chat function and had their camera turned off. A distress protocol was in place and a designated facilitator checked in on participants when required.

The online platform also helped overcome the logistic challenges of convening a group from different geographical location, people with clinical duties or childcare responsibilities. However, this together with the social media focused advertisement, may have led to digital exclusion.

A short video designed by the COMET group, explaining what a core outcome set is, [15] was shared with study participants before the focus groups. Based on pilot testing with our PPI advisory group, we used a case scenario to illustrate the concept of outcome. Nevertheless, the focus group discussions still focused on care experiences and processes. This issue was also encountered in other studies [16]. Through reflections after each focus group, the lead facilitator reframed participant’s views or experiences into follow up questions about outcomes.

#### Data analysis

The lead facilitator, who also analysed the data, is medically trained. This may have biased her views in favour of health care professional’s position, when interpreting themes relating to participant’s perceived quality of care. She also conducted the systematic literature search compiling outcomes reported in the literature. This exposure to the literature may have influenced how she labelled the outcomes identified in this study. However, these potential biases are counter balanced by her dual role of having lived experience of a long-term condition, the input of a second facilitator and data analyst with no medical training and no prior exposure to the literature search, and extensive involvement of the PPI advisory group in interpreting the data.

Previous qualitative studies in core outcome set development have used different analyses approaches, including thematic analysis, framework approach and approach informed by grounded theory [57–61]. Recent studies used interpretive evidence synthesis methods to transform participants’ experiences and views into measurable outcomes [62, 63]. We undertook thematic analysis using a data driven approach but also ensured we addressed the research question. The term ‘outcomes’ was used in a broad way to capture some aspects of experience that were important to stakeholders, even if they are not easily measurable with existing measurement tools, or do not fit with traditional medical understandings of ‘outcomes’.

When designing the Delphi survey, there was further deliberations with the multidisciplinary research team on combining outcome categories and labelling outcomes. Where possible, when preparing the plain English summary of the Delphi survey, we used the subthemes and words used by participants in this focus group study.

### Implications for future research

The outcomes identified in this focus group will inform the design of a Delphi survey to develop a core outcome set for future studies of pregnant women with multiple long-term conditions.

Future studies should explore ethnic inequality in pregnancy outcomes as suggested by one participant. Another participant suggested considering positive outcomes. This concept was previously discussed by Smith et al. who examined salutogenically focused outcomes of intrapartum interventions in systematic reviews [64]. The authors suggested shifting towards optimum or positive outcomes (health and wellbeing) instead of focusing on averting adverse outcomes [64].

Although we focused on research outcomes, the rich description of women’s experience of maternity care provided pointers of good clinical practice for health care professionals providing care to pregnant women with multiple long-term conditions and their children. This will be explored further in MuM-PreDiCT’s upcoming interview study on the experience of maternity care of pregnant women with multiple long-term conditions.

## Conclusions

Women with multiple long-term conditions emphasised outcomes related to the maternity care they received. Both women and their partners prioritised how better involvement in their care through enhanced communication and information sharing would help their experiences at different stages of pregnancy. These findings will inform the design of a standardised core outcome set for studies of pregnant women with multiple long-term conditions. However, women’s experiences also provide useful insights into broader ways in which maternity care for pregnant women with multiple long-term conditions can be improved without additional costs.

## Electronic supplementary material

Below is the link to the electronic supplementary material.

Supplementary Material 1

Supplementary Material 2

Supplementary Material 3

## Data Availability

All data generated or analysed during this study are included in this published article and its supplementary materials.

## List of Abbreviations

COMET: Core Outcome Measures for Effectiveness Trials
FG: Focus group
HCP: Health care professionals
PPI: Patient and public involvement
UK: United Kingdom
W: Women
