# Supplementary material for: Key outcomes for reporting in studies of pregnant women with multiple long-term conditions: a qualitative study
Source: BMC Pregnancy Childbirth. 2023 Aug 1;23:551. doi: 10.1186/s12884-023-05773-5 (PMC10391909; doi:10.1186/s12884-023-05773-5)
Supplement: Supplementary file 1 — Supplementary Material 1 [file 12884_2023_5773_MOESM1_ESM.docx]

**Supplementary Material 1: Focus group topic guide**

**1. Welcome**

Welcome everyone, facilitators and participants given opportunity to introduce themselves.

**2. What is a Core Outcome Set**

Before we start, I would like to share with you a 3 minute video that will explain what a Core Outcome Set is.[5]

**3. Aim of the focus group**

To find out **what outcomes you want researchers to measure** and report in all studies for pregnant women and birthing people with 2 or more long-term physical and mental health conditions.

The findings from this focus group will help us design the next stage of our study. It will be fed into surveys where we invite people to vote on which outcomes should be included in the final list of core outcomes.

**4. Housekeeping**

- The session will last **1.5 to 2 hours**.
- Please keep your microphone on **mute** when you are not speaking.
- To give everyone a chance to speak, please use the **raise your hand function** when you would like to share your views.
- Please feel free to use the **chat function** too to share your thoughts
- This is an open discussion, there is no right and wrong answers, and we want to hear the views of everyone here.
- Please **do not** discuss what your fellow participants **shared** in this focus group **beyond this session** today.
- We will start the recording shortly, all discussions will be **recorded** for analysis, you will not be identified in any publications of this research study.
- If at any time you would like to **avoid answering** a question, take a **break or leave** the focus group please do so.

**5. How to access support if you become distressed**

During the focus group, if you feel unable to continue with the discussion, please let one of our facilitators know so we can support you. If you feel comfortable to do so, you can switch off your camera, send a message to the facilitator. One of the facilitators can meet you in a separate link for some support.

Ask if there are any questions and clarify.

*Questioning/prompting by the facilitator is likely to include the following areas; exact wording will vary according to the flow of the conversation and what participants have already shared.*

**6. Discussions about outcomes**

Case scenario

Jane has multiple long-term health conditions. She takes multiple medications and sees her doctors regularly for her health conditions.

She becomes pregnant. Jane sees her doctors and midwives to plan for her pregnancy care.

Jane would like to know how her health conditions may impact on her pregnancy. Jane would also like to know how having her health conditions and being pregnant may impact on her health, her child and her family.

To answer her questions, Jane’s doctors and midwives look at previous studies on people like Jane who has multiple long-term conditions and became pregnant.

- If researchers study a large group of pregnant women with multiple long-term conditions like Jane, **what would you like** to see they **measure** in the study?
- If research found a new treatment / intervention / way of delivering care for people like Jane, what would be the **evidence that it worked**?
- What do you think researchers should measure to know any new treatment / change in care has **made a difference**?
- (If the discussion veers towards suggestions of how care can be improved, then ask: If we improve maternity care for people like Jane, what would you like to see **improve as a result**?)

**Remind participants the aim of the focus group before the discussion starts:**

- We are not asking about suggestions of how maternity care can be improved for pregnant women with multimorbidity, rather we are asking what the end results / ideal pregnancy or birth would look like if changes were made.
- We are interested in broad outcomes generic to all pregnancies with multiple long-term conditions, we are not focusing on outcomes specific to a particular health condition
- Outcomes for the following people: women and birthing people and their children
- Outcomes in the following time periods: pre-pregnancy, during pregnancy, immediately after pregnancy, longer term

**6. Closing remarks**

Do you have any further thoughts/ comments?

Thank you for participating in this focus group interview. Your input has been very valuable and will help us design the next stage of your study, which are surveys where people vote for outcomes, they feel are important.
