# Supplementary material for: Key outcomes for reporting in studies of pregnant women with multiple long-term conditions: a qualitative study
Source: BMC Pregnancy Childbirth. 2023 Aug 1;23:551. doi: 10.1186/s12884-023-05773-5 (PMC10391909; doi:10.1186/s12884-023-05773-5)
Supplement: Supplementary file 2 — Supplementary Material 2 [file 12884_2023_5773_MOESM2_ESM.docx]

**Supplementary Material 2: Participant flow chart**

Health care professionals

Expressed availability for the focus group and 1st invitation sent

N=10

Waiting list: expressed interest or availability after 1^st^ invitations were sent

N=3

Could not attend

(schedule clashes, illness or no response)

N=5

Participated in focus group

N=8

Expressed interest and eligible

N=19

Invited based on maximum variation sampling

N=18 women

Expressed interest and availability and were eligible

N=25 women

Could not attend

(schedule clashes, illness or no response)

N=6 women

Participated in focus group

N=12 women

Women
