# Supplementary material for: Key outcomes for reporting in studies of pregnant women with multiple long-term conditions: a qualitative study
Source: BMC Pregnancy Childbirth. 2023 Aug 1;23:551. doi: 10.1186/s12884-023-05773-5 (PMC10391909; doi:10.1186/s12884-023-05773-5)
Supplement: Supplementary file 3 — Supplementary Material 3 [file 12884_2023_5773_MOESM3_ESM.docx]

**Supplementary Material 3: Supplementary quotes**

| **Maternal outcome: before pregnancy** |
| --- |
| **Fertility**  “…your condition might make it very difficult to get pregnant and therefore getting pregnant is the achievement…” (Focus group [FG] 1, health care professionals [HCP] 4) |
| **Preconception care**  ***Uptake of preconception support***  “…It would be interesting to know…it's very rare for somebody to come in, purely for preconceptual counselling.” (FG1, HCP7)  ***Quality of preconception counselling***  “…it would be interesting to know are there certain conditions where the patients actually do get better counselling than other aspects.” (FG1, HCP7)  ***Preconception counselling on medications***  “…a lot of my medications is unknown...[Clinicians] recommend coming off it for a year before getting pregnant…if I have to stop this medication…my conditions may get so bad…I then might struggle to get pregnant…that could then take me a year or two…” (FG3, Women [W] 4) |
| **Maternal outcome: during pregnancy** |
| **Maternal death**  “…[my wife] dying…the pressure on her heart…I was very very scared…” (FG3, Partner [P] 2) |
| **Impact on long-term conditions**  “Anybody with a long term health condition knows the pregnancy is going to wreck your body...to have some statistics to say…on these medications, X number of women have found that this has happened in their pregnancy, will be a lot more reassuring...” (FG3, W1) |
| **Types of birth**  **Birth weight / macrosomia**  “…size was a big concern for us, especially because I’ve had wanted a vaginal birth but unfortunately ended up having… c-sections.” (FG3, W2) |
| **Miscarriage**  “…so like miscarriage, which I never thought of beforehand, till we started having regular miscarriages.” (FG3, P1) |
| **Birth injuries**  “I have [condition] so when it comes to injuries and wound it's so hard to heal and it takes a lot of time. And in the process of healing you may actually acquire another condition that was not there…the risk for injuries…should be measured, just to reduce injuries anytime to mother...” (FG2, W5) |
| **Interventions in pregnancy**  “…patients view a normal pregnancy has minimal intervention.…when we're trying to…achieve the best outcome for the mother and for the baby, by intervening with weekly appointments…the woman may feel that she's a failure…but it’s nobody’s fault, because obviously if we didn't intervene there was something horrific that happened…” (FG1, HCP7)  ***Limited options***  ***Analgesia***  “…term birth, via a normal delivery with no monitoring, minimal analgesic…there is some impacts of their disease…that means, that some of those choices aren't open to them.” (FG1, HCP4) |
| **Haemorrhage**  **Blood pressure**  “… the type of birth, the pain relief, the blood pressure. Any haemorrhaging...” (FG1, HCP2) |
| **Maternal outcome: after pregnancy** |
| **Postnatal and long-term care**  ***Length of postnatal support***  “…it's considering the kind of home care…support packages that we put in place… women are going home, on their own, or may have a partner, and feel the extra pressure…how that support is going to continue beyond the pregnancy element of it.” (FG1, HCP5)  ***Postnatal support for raising a child***  “…you need the support, after you've had the baby…your [multiple long-term] conditions, I’m guessing, are probably going to be all over the place…to raise the baby when you're going to need to be up all night and feeding, but actually you still got to care for yourself…” (FG3, W3)  ***Quality of postnatal support***  “But also postnatal [check]… it varies…is there a way of evaluating how efficient that might be….we are the last people to actually see these women…” (FG1, HCP7)  “…it's not just stopping at birth. It's then the follow up…are you being followed up properly, and when you’ve left the hospital, again is that information being passed through, about your kind of health needs.” (FG2, W4)  ***Emotional support***  “…definitely emotional support…I just found that in my pregnancy…it was quite hard because…I gave birth quite early [baby] was premature…” (FG2, W6)  “…my hospital stay and all was quite traumatic and happened very quickly…that all hit me more after I had the baby and…he was safe…abit of a check in at some stage…would be a good thing to consider for people with multiple health condition...” (FG3, W4)  ***Support for family***  “…the community midwives were coming out, we were having hour long meetings…there still wasn't time to cover what happened to [husband]…to [child]. It was mainly focused on me…that is definitely something that needs to change, it needs to be looked at the family unit as a whole...” (FG3, W2) |
| **Perinatal mental health**  ***Postnatal depression***  “…postnatal depression…we didn't know where to go, what support was available…I’ve heard people still don’t pick up depression… with ethnic minorities and Asians its quite a taboo subject…” (FG2, W6)  ***Impact on pre-existing mental illness***  “…the high risk period…in women's mental health cycle…from 36 weeks pregnancy to about six weeks afterwards, so we could look at that period and look at rates of relapse…” (FG1, HCP8)  ***Emotional and mental wellbeing***  “… the trauma of being in hospital and having the baby and having the baby taken away, I probably found it more difficult in the weeks after...” (FG3, W4)  ***Impact of mental health on physical health***  “…something like depression, they will have an effect if they got other…physical problems, they might get so depressed and actually have very poor compliance with the treatment.” (FG1, HCP7)  ***Impact of physical health on mental health***  “… how has my mental health regarding my diabetes changed because, when I’m pregnant…I get really, really scared of having high blood sugars because it's so drilled into you how dangerous it is.” (FG3, W2)  ***Experience of perinatal mental health support***  “…if you’ve got multiple health conditions…our brains are already swirling with a million different things, add in to that the anxiety of pregnancy and childbirth and…the unknown…the fact we're not being checked in on.” (FG3, W1) |
| **Ability to breastfeed**  “I wanted to breastfeed straight away, I was told I couldn't…because of the [pregnancy complication]…” (FG3, W2) |
| **Breastfeeding support**  …I wanted help with the breastfeeding...But I didn't get that... sometimes I used to cry because I can't do it... But you just didn't feel like you got the support…someone being there and helping you and saying it's okay…there's breast pumps available…it takes time….” (FG2, W6) |
| **Establishing feeding**  **Engaging with healthy behaviour**  “…if their mental health is destabilized they are less likely to engage in positive health behaviours…requirements at different stages of pregnancy…potential disruption to mum, to baby, to the bond…establishing feeding and bonding.” (FG1, HCP 8) |
| **Pressure in maternal role**  “…there’s lots of pressure on mothers to be the perfect mother and, about breastfeeding…” (FG1, HCP7) |
| **Maternal guilt**  “…there's an awful lot of guilt isn't there, when you do have mothers if there's multiple morbidities or the effects of their [medications] or their illness on their child.” (FG1, HCP 7)  “I felt really guilty... that it was because I had health conditions that [my child] had a problem…So my child is like [x] months now and…I still think about that quite a lot… nobody really spoke to me about that after…” (FG2, W4) |
| **Parent and infant bonding**  “…[child] has always been a lot more independent…we felt that was the result of having been in special care [when he was a newborn]…he had been away from us for so long…how do we support building a better bond with their parents…” (FG3, W2)  “…if a daddy's really struggling with how the baby arrived then…that's going to put up barriers to bonding with the new baby.” (FG3, W4) |
| **Skin-to-skin**  “I think outcomes for babies are things like, was skin to skin preserved, was the golden hour observed as much as possible, how much contact was given between parents and babies, even if baby was in special care...” (FG3, W2) |
| **Recovery time**  “…even after a C-section…for a healthy person…it's a six week recovery, it’s a major surgery. Put in there all the additional needs, and we have no idea what's going to go on…the services…offered aren't long enough.” (FG3, W1)  “…with her [health conditions]…will she be able to recover as fast as normal…” (FG3, P1) |
| **Development of new health conditions**  “…at some point in my life, maybe I’m likely to develop type two diabetes... deep vein thrombosis you're more likely to clot…that information hasn't been shared…Like if you've had a pregnancy and you're overweight or have a higher BMI (body mass index), and you have certain conditions. They should have a checklist that you could say screen for this...” (FG2, W3) |
| **Long-term cardiovascular outcomes**  “…in terms of long term outcomes for their risk of developing strokes, MIs (myocardial infarctions)…later on, if they've had really intensive help or support in managing their conditioning well during those nine months, whether that could have a positive outcome on their future long term…cardiovascular health…outcomes.” (FG1, HCP1) |
| **Maternal outcome: all stages of pregnancy** |
| **Quality and experience of care**  “…like the GAD-7 (a measuring tool for mental health) where we’re talking about mental health, actually about the full experience of pregnancy being looked after under that health profession that would be really quite useful.” (FG3, W1) |
| **Change in medication**  “…if you take this, then you gotta give it up… for…years…we have that with one [medication] that you could have been given, but …we got to wait an entire length of time till it is out of your system…” (FG3, P1) |
| **Holistic care / multidisciplinary coordination of care**  “…when I came from general medicine into obstetric medicine, it sometimes felt like things were like poetry in motion when someone got pregnant, because you had all of these services coming together to help them. So, if they had long standing diabetes they'd suddenly have three or four different people supporting them.” (FG1, HCP1) |
| **Shared care decision**  “The measurement of does that woman feel as though she's a partner in that care…it's about us not being paternalistic…does she feel as though she was involved?” (FG1, HCP2)  “…the lactation consultant comes to me and says, well, you need to hand express because your baby who's in neonatal intensive care needs breast milk…she didn't discuss the benefits…So I was sort of forced into it. No choices.” (FG2, W3) |
| **Continuity of care**  ***Information being passed on***  “…what's really important for [women] is the continuity in the transfer of information between those different stages…at certain stages, they get a lot of support and… communication, but…that doesn't happen naturally as they transition back to…general medical services...” (FG1, HCP4)  ***Seeing the same health care professionals***  “…my main concern is, if you introduce me to Dr so and so… am I ever going to see Dr so and so, again...” (FG2, W1) |
| **Social and peer support**  “…I find myself … searching the Internet for blogs and anything of women who had the same conditions as me who had gone through pregnancy…it's like finding a needle in a haystack…and when I did find anything, I was like, oh yes that is how I feel.” (FG2, W4)  “…consider other clubs and groups because nowadays there's a lot of mum groups …whatsapp groups...” (FG2, W6)  “…having someone who has the same condition as you, being part of the journey, would be a) they can advocate for you when really tricky subject comes up; b) they can take a lot more in because if you're getting shocking news at that time, your focus is not going to be on what the doctors or healthcare providers are talking about; and c) just having someone who has been through it, makes a whole lot of difference.” (FG2, W1) |
| **Information provision for preparedness**  ***Informed of potential risks***  “…doctors always like trying to make it as nice and cushy as possible…but they're not honest with you about the possible things that could happen.” (FG2, W3)  “…I hate being taken by surprise…if I don't know what's coming, then I feel really, really out of control.” (FG3, W2)  ***Informed of care***  “…how informed they felt, how kept in the picture they felt even in emergency situations…” (FG2, W2)  “When your baby…go on to CPAP (continuous positive airway pressure)…seeing your baby with this big mask…it's the scariest thing…had I been warned that my child would look like that, maybe I would have felt better…” (FG2, W3)  ***Informed of support / services available***  “People who have multiple medical conditions and planning for pregnancy/baby need more support, we don't know where to get that support and help...” (FG3, W6)  ***Informed for self care***  “…how can I adapt anything first…how can I adapt routines to help…knowledge is power…we can then sort them out ready for when mother and baby come home.” (FG3, P1) |
| **Birth experience**  “For me I think the biggest thing would be…birth satisfaction… we had a very, very detailed birth plan…[the health care team] didn't speak to my consultant…it went almost as wrong as it could have gone.” (FG2, W2) |
| **Accessibility of services**  ***Physical barriers***  “…I ended up having to spend a night, in an inaccessible room…my wheelchair had to be kept outside…I was literally stuck in the bed…” (FG2, W2)  ***Social barriers***  “…I ended up being assigned to a specialist health visitor…who purposely did do home visits, because domestic violence survivors can't go to a lot of the places they had previously gone to…”(FG2, W2)  ***Communication barriers***  “…several friends of mine who are deaf BSL [British Sign Language] users…they had to have their hearing parents, come along for the birth, because the hospital told them they couldn't even get an interpreter.” (FG2, W2)  ***Travel distances***  “…I’m not going to travel really far when I’m pregnant…if I need urgent care, I don’t have the choice of travel unfortunately.” (FG, W3) |
| **Health care professionals’ knowledge and skills**  “… how confident the team is…to be able to care for us…It’s their knowledge that’s more important. (FG3, W1 and P1) |
| **Health care professionals’ knowledge of the women**  *“…*at every appointment… if I’m introduced to a new [health care professional], I say, have they read my notes before…I want them to be knowledgeable about me… how am I going to trust you to see me through the hardest point in my life, so much pain, so much stress, if I can't even trust that you know who I am*.”* (FG2, W1) |
| **Health care professionals’ attitude towards the women**  “…if the baby does have an issue and requires specialist treatment, how is that mother… who has multiple health conditions…treated...When they were talking about my little boy, they would often say oh he's the baby of the [name of health condition] mother with other health conditions. And then you could almost see the other nurses going hmm.” (FG2, W4)  “…they said, how are you going to manage to look after your child…because disabled women are seen as…not basically being suitable for having children that we just get completely bypassed. And my needs weren't met... And it's down to the attitudes of doctors, beyond anything else.” (FG2, W2)  “… I was discriminated and judged…I could not pick up my baby, no one was coming in to look at her, feed her, change her, nothing. I was in a room by myself…I felt like they have put a hazardous sign on my door…” (FG2, W1) |
| **Hospital’s facilities / services**  “…you choose the best hospital to meet your care for that day… the most [complete ones] for when you go in for tests, checkups and also for the main day of the birth, where things can go wrong, and you want the specialists to all meet…”(FG3, P1) |
| **Personalised care**  “…Are those discussions being had between the doctor and the patient, or are they just following the guidelines…[For postnatal care]…it was again almost like there was a sheet of guidelines that were being read off and ticked…having that a bit more individualized care, particularly for moms who have multiple health conditions.” (FG2, W4) |
| **Consistency of care**  “…the other thing that gets to me is… the consistency between…health workers…My [child] was born early…they work on an actual uncorrected age…my friends are getting one set of treatment…but I don't get [the same treatment]…if there's a guideline of what they need to do…if it's all the same for everyone...” (FG2, W3) |
| **Impact on partner**  ***Partner’s caring role***  “[my partner] is my carer, he has to physically help me get up and get dressed… The stuff that he has to see now that I’m sure it's traumatized him already…let alone a difficult birth or a birthing plan that goes wrong.” (FG3, W1)  ***Support for partner***  “…because there are times, where I feel like my husband has needed more input than I have. But he's just never been offered it...” (FG3, W2)  ***Involvement of partner***  “…the male is supported as much as the female, the male is included in all of the information, the males not felt that they can't ask questions…”(FG3, W1)  ***Partner’s mental well being***  “…it would be good for fathers to be involved…like how it impacted them. Because I know, during my labour, [my partner] had a really difficult time…there were so many machines going on…so many doctors and anaesthetists…he would still talk about it now, how scary it was...” (FG3, W4) |
| **Expectation of care and outcomes**  “…the expectation of what she wanted for herself for that care…the outcomes…what is important to her…Because, sometimes, we…measure it according to our expectations...” (FG1, HCP2)  “…it's really difficult with multiple long term conditions in pregnancy…the outcomes are so wide… I may recommend you not to get pregnant, because your risk of dying is so high, you may choose to get pregnant, but the satisfaction should be that I support you through that journey and you felt supported, whether your expectation has been met...” (FG1, HCP4) |
| **Compliance with medication**  “…interesting…to…find out…how many women have actually stopped taking their medication…” (FG1, HCP7)  “…we often see women who have, stopped their medication automatically as soon as they find out they're pregnant…then explaining to the woman the [impact their untreated] illness could have on the baby, and the pregnancy.” (FG1, HCP8) |
| **Quality of life**  “…you might want to know the impact of the multiple morbidities on her quality of life...” (FG1, HCP3) |
| **Children’s outcome** |
| **Timing of birth (preterm birth)**  **Baby’s growth**  **Birth defect**  **Child’s death**  “… I'd like to see the outcomes to the birth, what measurements would be for the growth of the baby, stillbirth, outcomes, abnormalities…maybe even type of births, the timing of the birth.” (FG1, HCP2)  “… the usual stats that are covered regarding…maternity care…maternal mortality and infant mortality…” (FG2, W2)  “…any kind of birth…defect statistics…” (FG3, W1) |
| **Separation of mother from newborn baby**  “…I gave birth to my [child], and she was rushed in to the neonatal intensive care unit because of her gestation…I was having nightmares that my child was looking for me and couldn’t find me… it's very traumatic…you should be warned about it… to put you in a maternity ward with the other mothers that have just had their babies and they are so happy and excited and it's the first night. And here you are…babyless.” (FG2, W3) |
| **Baby’s lung development (respiratory distress syndrome)**  “…my little boy had problems with his lungs, they hadn’t quite developed, which is something that's linked with my [condition] that… I wasn't aware of…and also…delivering a little bit early and having a section, they kinda all added up to, leading to that.” (FG2, W4) |
| **Infant mental health**  “…there's quite a lot of research about infant mental health, quality of the [mother baby] relationship impacting on, development of the baby and how the absence of that relationship can have a negative impact…” (FG1, HCP8) |
| **Impact of medication in pregnancy**  **Baby’s condition at birth**  “I had tried to search for more information on the Internet and had consulted with doctors and other people with the same condition around medication, because I wanted to be sure that it was safe, particularly for the baby…” (FG3, W5)  “We need education about pregnancy and medication we are using and their side effects from our doctors.” (FG3, W6)  “…how the baby could potentially be whenever they were born…that was a massive concern of mine…with all the… extra medications that I was taken during the pregnancy.” (FG3, W4) |
| **Neonatal intervention**  “…if you're looking at what interventions babies have had, hopefully that cascade of interventions that happens when a baby is sick, would be more considered [by health care professionals].” (FG3, W2) |
| **Inheritance of mother’s condition**  “What will happen in my next pregnancy, will my next child be diabetic or something, how will I manage it, and everything is still a worry.” (FG2, W5)  “…I was saying to my [child] to get that blood test, because sometimes it's inherited…so it can be like family conditions…they should definitely screen through when they do your medical history...” (FG2, W6) |
| **Developmental outcomes**  “…we often use, again probably not perfect in any shape or form…two year old neurodevelopmental outcomes.” (FG1, HCP6) |
| **Metabolic syndrome**  “[regarding outcomes in children]…inflammatory diseases and the effect on metabolic syndrome, in the long term, also neurodevelopmental conditions in the longer term.” (FG1, HCP1) |
| **Neonatal morbidity**  “…so the morbidity would be typically looking at…whether they require intensive care, support in the neonatal period, whether they develop respiratory distress, and or depending on the condition, some of those might have an adverse impact on the cardiovascular function of the baby or the metabolic controls...” (FG1, HCP6) |
| **Participation in society**  “…some of the long term outcomes which may be more relevant as to how that child is going to be... be functioning as an adult…to the society as he or she grows.” (FG1, HCP6) |
| **Health care utilisation** |
| **Admission to neonatal unit**  “…they don't give you the possibility of [your newborn] going to neonatal intensive care unit (NICU)...(FG2, W3).” |
| **Number of appointments**  “…my appointments they've made sure…I won’t go in on a Monday and then go in on a Wednesday... They tried to have one appointment, at the right time in my pregnancy, so that I see everybody on that day…(FG2, W1).” |
| **Length of hospitalisation**  “so what they want is some measure of…what is … the length of stay going to be, how long is my care going to need to be in hospital after I have given birth...” (FG1, HCP4) |
| **Number of hospital admission during pregnancy**  **Financial implications**  “…how many times during her pregnancy she requires admission to hospital? Does she require time off of work?” (FG1, HCP3)  “…the financial impacts for them that are very worrying, additional visits…” (FG1, HCP4) |
| **Consideration for future studies** |
| **Impact of ethnicity**  “…looking at what impact it would have on them specifically, i.e. from a black or brown perspective…” (FG1, HCP5) |
